# Alloreactivity of Virus-Specific T Cells: Possible Implication of Graft-Versus-Host Disease and Graft-Versus-Leukemia Effects

**DOI:** 10.3389/fimmu.2013.00330

**Published:** 2013-10-14

**Authors:** Shigeo Fuji, Markus Kapp, Hermann Einsele

**Affiliations:** ^1^Department of Internal Medicine II, Division of Hematology, University Hospital of Würzburg, Würzburg, Germany; ^2^Division of Hematopoietic Stem Cell Transplantation, National Cancer Center Hospital, Tokyo, Japan

**Keywords:** allogeneic stem cell transplantation, virus-specific T-cell, GVHD, HLA antigens, GVL, mismatch

## Abstract

Immune reconstitution of functional virus-specific T cells after allogeneic hematopoietic stem cell transplantation (HSCT) has been intensively investigated. However, the possible role of crossreactivity of these virus-specific T cells against allogeneic targets is still unclear. Theoretically, as in the field of organ transplantation, virus-specific T cells possess crossreactivity potential after allogeneic HSCT. Such crossreactivity is assumed to play a role in graft-versus-host disease and graft-versus-leukemia effects. In this article, we aim to give a comprehensive overview of current understanding about crossreactivity of virus-specific T cells.

## Introduction

The reconstitution of functional virus-specific T cells and the importance of these T cells in the control of viral diseases following allogeneic hematopoietic stem cell transplantation (HSCT) has been extensively investigated (1). This led to a successful transfer into the clinical setting within adoptive immunotherapeutic approaches (2).

Among various viruses, immune reconstitution against cytomegalovirus (CMV) has been most intensively studied. Regarding the recovery of CMV-specific T cells after allogeneic HSCT, several reports showed that subclinical CMV antigenemia drives the reconstitution of functional CMV-specific T cells (3–5). The rapid homeostatic expansion of CMV-specific T cells suggests that such T cells might be largely memory T cells, considering the insufficient regeneration of T cells due to the poor thymic function early after allogeneic HSCT. Similar to the immune response after primary CMV infection (6, 7), the proportion of virus-specific T cells including CMV-specific T cells can be high and in some cases CMV-specific T cells make up more than 10% of the circulating T cells after allogeneic HSCT (8, 9). If the number and/or functionality of CMV-specific T cells is insufficient in recipients of an allogeneic stem cell graft, they are at high risk of persistent viremia and CMV disease (10, 11).

Although immune reconstitution of virus-specific T cells has been intensively studied, the fact of possible alloreactivity of virus-specific T cells in the allograft recipient has only been evaluated in few trials (12, 13). However, as suggested in other fields like organ transplantation, virus-specific T cells are assumed to play a role in alloreactivity similarly in the field of allogeneic HSCT (14, 15).

In this review, we focus on crossreactivity of virus-specific T cells against allogeneic targets, and discuss the possible implication of such reactions on the allogeneic immune responses after allogeneic HSCT.

## Crossreactivity of Virus-Specific T Cells

Virus-specific T cells, which dominate the memory pool in humans, have been reported to have the potential of crossreactivity (14). Crossreactivity of T cells is the ability to recognize several different peptide/human leukocyte antigen (HLA) complexes. CD4^+^/CD8^+^ and naïve/memory T cells were shown to mediate crossreactivity against allogeneic targets (16, 17). Upon activation, memory T cells proliferate more quickly and produce more cytokines than naïve T cells (18, 19). Considering these rapid and vigorous T-cell responses mediated by memory T cells when compared to naïve T cells, one could assume that such alloreactivity of virus-specific memory T cells could play a role in the pathogenesis of early-onset acute GVHD, in particular hyperacute GVHD, following HLA mismatched HSCT. The difference of these T cells in the ability to expand and express cytotoxic molecules might also contribute to the difference in the outcome in patients with acute GVHD. Virus-specific T cells might have much higher avidity against allogeneic targets after HLA mismatched HSCT when compared to that after HLA-matched HSCT, considering the mechanism of negative selection in thymus (20, 21). Virus-specific T cells possessing high avidity against autologous HLA molecules with a self-peptide should originally be deleted in thymus. However, if virus-specific T cells recognize the complex of a peptide and non-autologous HLA molecule via their T-cell receptor, the avidity of T cells against this complex can be high because this HLA molecule is not expressed in the thymus and thus cannot induce the negative selection in the thymus (22). One hypothesis could be that, if such strong peptide-specific crossreaction exists against tumor-associated antigen (TAA)-derived peptides expressed in leukemia cells accidentally, it should lead to a strong graft-versus-leukemia (GVL) effect. However, there is no data available so far which could support this idea. Furthermore, crossreactivity against non-self HLA presenting a non-polymorphic hematopoietic cell-specific peptide or TAA-derived peptide might provide therapeutic tools for immunotherapy, similar to the concept for minor histocompatibility antigens like HA-1.

In addition, virus-specific T cells which have weak to moderate avidity against autologous HLA molecules with a self-peptide might theoretically remain *in vivo*, considering the mechanism of positive selection in thymus. Such virus-specific T cells might subsequently exert alloreaction in the setting of HLA-matched HSCT, only when the strong activating signals by various cytokines stimulate them (20, 21). However, such crossreactivity by virus-specific T cells against autologous HLA molecules has not yet been demonstrated so far.

Regarding crossreactivity of virus-specific T cells, Epstein–Barr virus (EBV)-specific T cells have been studied in detail (14). Burrows et al. demonstrated crossreactivity of EBV EBNA3A-specific T-cell clones for the immunodominant peptide FLRGRAYGL presented on HLA-B*08:01 against the alloantigen HLA-B*44:02 (23). This finding was reconfirmed by other researchers (24, 25). These reports did not demonstrate the requirement of a specific peptide presented on HLA molecule for crossreactivity. Later on, it has been demonstrated that crossreactivity of this EBV EBNA3A-specific T-cell clones is dependent on the presentation of the self-peptide derived from the ABCD3 gene (EEYLQAFTY) (26). Here, we have to point out the significant difference between the two peptides’ sequences, suggesting that crossreactivity does not necessarily require a homology in sequences indicating promiscuity of the T-cell receptor. Another EBV EBNA3A-specific T-cell clone, recognizing the complex of HLA-B*08:01 and an EBNA3A-derived peptide FLRGRAYGL, has been shown to react with the complex of HLA-B*35:01 and the self-peptide derived from Cytochrome P450 (KPIVVLHGY) (22). This study demonstrated a similar avidity of the EBNA3A-specific T-cell clone against the complex of HLA-B*08:01 with EBNA3A-derived peptide and the complex of HLA-B*35:01 with Cytochrome P450-derived peptide.

Regarding other viruses, Amir et al. reported that crossreactivity of virus-specific T cells against mismatched foreign allogeneic HLA was common (25). They used expanded T cells which were isolated using various combinations of tetramers loaded with a virus-derived immunogenic peptide. The target cells were a panel of lymphoblastoid cell lines (LCL) expressing various combinations of HLA molecules. A major finding was that a large number of virus-specific T-cell clones have crossreactivity potential against various HLA molecules. It is also important that some CD8 T-cell clones showed crossreactivity against HLA class II, even though most CD8 and CD4 T cells were crossreactive against HLA class I and class II molecules, respectively (25). A similar phenomenon showing the recognition of HLA class II by CD8 T cells was also reported by Rist et al. (27).

However, in a clinical trial using expanded virus-specific cytotoxic T-cell lines for the treatment of viral diseases, GVHD was rarely (6.5%) observed even when crossreactivity of expanded virus-specific T cells was observed *in vitro* (13). The fact of rarely observing GVHD clinically in expanded virus-specific T cells in this study might be caused by the absence of the correct crossreactive HLA molecule in the mismatch combinations, the difference in homing capacities and the lack of respective target molecules on the GVHD-target organs. Therefore, the clinical relevance of crossreactivity which was detectable *in vitro* should be further clarified in clinical trials.

Previously, it has been assumed that crossreactivity against allogeneic HLA is independent of the peptides in the HLA groove but that the allogeneic mismatched HLA molecules are the target of this cross-reactivity. In contrast, recent reports support the idea that crossreactivity against allogeneic HLA is peptide-dependent as reviewed previously (28). Actually, it is still difficult to demonstrate non-peptide-dependency experimentally, because even transporter-associated with antigen processing (TAP) deficient cell lines, which were believed to be completely deficient in antigen processing- and antigen presenting-capability, are able to load endogenous peptides on HLA molecule (28). Weinzierl et al. have demonstrated the presentation of many peptides by HLA molecules on the TAP-deficient cell line (29).

Peptide-specificity of alloreactive T cells is also supported by tissue/cell type-specific alloreactivity of clinical samples. Various reports showed the presence of tissue-specific alloreactive T cells in patients with graft failure after organ transplant (30– 33). Deckers et al. have reported that the cytotoxic potential of graft-infiltrating CD8^+^ T cells against proximal tubular epithelial cells (PTEC), gonadal vein endothelial cells (GOVEC), and splenocytes depends on the clone of graft-infiltrating CD8^+^ T cells in renal allografts (30, 31). In this report (30), 46 graft-infiltrating CD8^+^ T cells were cloned. Out of 46 clones, 7 lines recognized PTEC but not splenocytes derived from the same donor. Thirty lines recognized PTEC and splenocytes equally. One line preferentially recognized splenocytes over PTEC. Eight lines were not cytotoxic either to PTEC or to splenocytes. Therefore, each clone recognized different targets of the recipient. Jutte et al. also showed the specific cytotoxicity against heart endothelial cells by expanded graft-infiltrating T cells in heart allografts (32, 33). Other reports using virus-specific T cells also support the idea of cell type-specific crossreactivity of virus-specific T cells. For example a VZV-IE62-specific HLA-A2 restricted T-cell clone recognizes allogeneic HLA-B*57:01-expressing LCLs, phytohemagglutinin (PHA) blasts, and monocyte-derived dendritic cells (DCs), but does not recognize HLA-B*57:01-expressing B-cells, T cells, monocytes nor fibroblasts in a standard ^51^Cr release assay. Such tissue/cell type-specific crossreactivity has been also reported by D’Orgogna et al. showing that allogeneic HLA-B*44:02-positive PTECs and human umbilical vein endothelial cells (HUVECs) are poor targets for EBV EBNA3A-specific T cells due to the lack of EEYLQAFTY peptide presentation (34). Amir et al. also reported that certain cell types with the correct HLA mismatch were recognized by virus-specific T cells while other cell types were not (25).

Regarding T cells in a patient with acute GVHD after HLA mismatched HSCT, single-peptide specificity was documented using a single small hairpin RNA (shRNA) system (12). There is no data available for chronic GVHD. The concept of tissue-specific alloreactivity also might be applied to GVHD following allogeneic HSCT which is also restricted to a few organs (especially skin, gut, and liver), even though other factors such as proinflammatory environment caused by the conditioning regimen affect the specificity of target organs. Tissue damage could change the expression of genes as well as the expression of HLA molecules. Furthermore, the effects of cytokine/chemokine are expected to differ among different organs.

## Clinical Data Suggesting Alloreactivity and Viral Infection

Over the last decades, various retrospective studies have shown the possible association between viral infection and graft rejection/GVHD (15, 35). Although there is no published data assessing the impact of CMV prophylaxis on the incidence of GVHD, prospective studies assessing the impact of CMV prophylaxis using ganciclovir in organ transplantation have demonstrated a reduced risk of graft rejection in the group receiving prophylactic CMV therapy, which supports the idea that CMV infection can be associated with an increased risk of graft rejection (35).

One recent report evoked the attention of researchers on the importance of CMV infection regarding the effects on the GVL effect in allogeneic HSCT (36). In this study, Elmaagacli et al. demonstrated a significant association between early CMV reactivation and a reduced risk of relapse in acute myeloid leukemia (AML) patients after allogeneic HSCT. Patients who developed early CMV replication detected by pp65 antigenemia assay had a significantly lower risk of relapse compared with those without early CMV replication. Another group reported a similar finding in patients with chronic myeloid leukemia (CML) (37). A very recent report from Fred Hutchinson Cancer Research Center also supported the hypothesis that early CMV reactivation in AML but not in other diseases including CML may be associated with a reduced risk of relapse, even though the impact was much less in this study compared to the previous report (38). Regarding the difference among the diseases, it is potentially due to the difference in epitopes expressed on HLA molecules. The identification of target molecules recognized by virus-specific T cells might give us a clue to this issue. In contrast to these trials (36–38), persistent CMV antigenemia was associated with a poor clinical outcome possibly due to the fact that the impaired immune status is also associated with an insufficient GVL effects by functional T cells (39).

Previously, Parkman and colleagues reported that the presence of immune response to herpes viruses was associated with a reduced risk of relapse in patients with acute leukemia after cord blood transplantation, which led to a better progression-free survival (40). In this study, there was no association between the absolute count of lymphocytes and the presence of an antigen-specific immune response. Interestingly, neither acute nor chronic GVHD had any significant impact on the likelihood of leukemic relapse, suggesting that virus-specific T cells specifically induced GVL effects and graft-versus-host reaction. Hoegh-Petersen et al. also showed the significant impact of herpes virus-specific T cells at 56 days after HSCT on the incidence of subsequent relapse (41). In this study, in patients without relapse, functional T cells against various viral antigens including BZLF1 and EBNA3 were detected. There is a possibility that anti-viral immunity may be just a surrogate factor for the immunocompetence of the recipient after allogeneic HSCT and thus not have a direct causal relation with GVHD/GVL but being an epiphenomenon of other factors such as inflammation, cytokine storm, and so on. Thus, more detail about the crossreactivity of virus-specific T cells should be clarified in the setting of allogeneic HSCT. A better understanding of their role in alloreactivity will help to reduce acute and chronic GVHD but also to mediate the important GVL reactivity by more sophisticated immunosuppressive strategies, which makes allogeneic HSCT still the most effective form of immunotherapy – allowing to cure patients with hematological malignancies which are incurable by any other form of treatment.

## Conclusion

**Figure 1:**
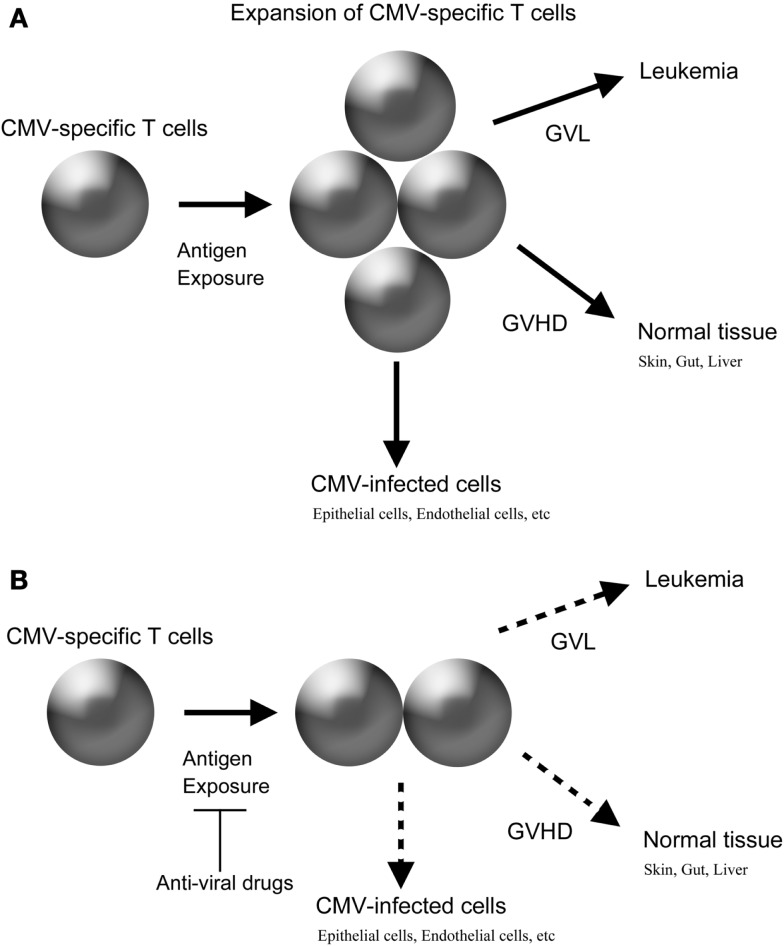
**Possible crossreactivity of CMV-specific T cells in allogeneic HSCT and the impact of anti-viral drugs**. **(A)** Without anti-CMV drugs. **(B)** With anti-CMV drugs.

Virus-specific T cells can recognize and target allogeneic HLA in a peptide-dependent manner. In an HLA mismatched HSCT, the avidity of such crossreactivity can be theoretically high enough to exert clinically meaningful alloreaction. Furthermore, in an HLA-matched HSCT, there is a possibility that virus-specific T cells develop alloreaction, even if virus-specific T cells have only low to intermediate avidity against autologous targets, considering the high frequency of virus-specific T cells and the unique milieu of cytokine storm after a conditioning regimen. Furthermore, intensive prophylaxis of virus infection after allogeneic HSCT might be beneficial to reduce the incidence of GVHD similar to that after organ transplantation because such intervention could reduce the amount of antigen exposure, which is expected to decrease the expansion of donor-derived virus-specific T cells (Figure 1).

In conclusion, elaborate basic and clinical research to clarify the detail of crossreactivity of virus-specific T cells after allogeneic HSCT is warranted.

## Conflict of Interest Statement

The authors declare that the research was conducted in the absence of any commercial or financial relationships that could be construed as a potential conflict of interest.
